# Pedobarographic Analysis Following Pemberton's Pericapsular Osteotomy for Unilateral Developmental Dysplasia of the Hip: An Observational Study

**DOI:** 10.1097/MD.0000000000000932

**Published:** 2015-06-12

**Authors:** Chao Xu, Ya-Bo Yan, Xiong Zhao, Xin-Xin Wen, Lei Shang, Lu-Yu Huang, Wei Lei

**Affiliations:** From Department of Orthopaedics, Xijing Hospital (CX, YBY, XZ, XXW, LYH, WL); and Department of Health Statistics, Faculty of Preventive Medicine, the Fourth Military Medical University, Xi’an, PR China (LS).

## Abstract

Successful clinical and radiographic outcomes have been reported in patients with unilateral developmental dysplasia of the hip (DDH) following Pemberton pericapsular osteotomy (PPO). However, residual gait deviations are seen in both the affected and unaffected limbs. To date it is not known whether these deviations result in abnormal plantar pressure in such patients.

This study investigated this possibility by performing pedobarographic, clinical, and radiographic examinations after PPO in 20 patients (age: 102.5 ± 19.0 months) with unilateral DDH who underwent PPO at 34.2 ± 9.8 months of age. Plantar pressure was evaluated using the Footscan pressure plate (RsScan International, Olen, Belgium). Each foot was subdivided into 10 zones and peak pressure, force–time integral as a percentage of total FTI, and contact time as a percentage of total stance time was estimated. The minimum duration of follow-up was 24 months (mean: 68.3 ± 20.3 months). The data were compared with 20 age- and weight-matched healthy controls.

Despite clinical and radiographic examinations showing satisfactory results according to modified McKay and Severin criteria, significant differences in plantar pressure parameters were identified between the affected limbs, the unaffected limbs, and normal controls. No significant differences were found between patients classified as “excellent or good” and those rated as “fair” according to the modified Severin criteria.

Pedobarographic results showed the existence of the residual plantar pressure deviations during walking in patients treated with PPO for unilateral DDH. Longer follow-up will be needed to more fully evaluate the effect of these deviations on gait.

## Abstract 2

Supplemental Digital Content is available in the text

## INTRODUCTION

Developmental dysplasia of the hip (DDH) comprises a series of structural abnormalities of the hip joint, ranging in severity from mild dysplasia to frank dislocation.^1^ The incidence of DDH is between 0.15% and 2%,^2,3^ affecting predominantly girls in a ratio of about 5–8:1.^4^

In affected patients, dislocation or dysplasia of the hip can arise following a normal neonatal screening examination. Unless the DDH is diagnosed and treated in time, it may result in a severe disability. Harris^5^ suggested that the upper age at which hip reduction would result in satisfactory acetabular development was 4 years. If the nonsurgical methods are ineffective, surgical treatment is required.

Pemberton pericapsular osteotomy (PPO) was defined by Pemberton^6^ in 1965 and is widely recognized as a safe and effective procedure both in terms of clinical and radiographic outcomes in children.^7–11^ However, there are reports of residual gait deviations in patients previously treated with PPO for DDH that decreased function and made revision surgery challenging.^12,13^

Previous gait studies following PPO and other surgical interventions for DDH have primarily focused on the kinematics and kinetics of the main joints of the lower extremity or on gait patterns.^12–15^ These studies indicate that both the affected and unaffected hips were subject to higher loading and high loading rates during level walking when compared with healthy controls.^13^ The increased loading was associated with increased risk of avascular necrosis of the femoral head^16,17^ and degenerative hip osteoarthritis.^18^ A trend toward greater walking velocity and an improvement in stride length were noted by Karam et al^15^ together with a decrease in mean total daily steps. Another study in 9 female subjects reported a reduced flexor moment in the hip joint of the affected limb.^14^ As we known, the changes in gait probably affect the pressure distribution under the foot.^19^ However, to our knowledge, no study to date has investigated plantar pressures after PPO for unilateral DDH.

This study investigated whether plantar pressures remained normal in patients following successful PPO treatment for unilateral DDH. Our hypothesis was that patients would achieve satisfactory clinical and radiographic outcomes, but would still manifest residual deviations in pedobarographic parameters in comparison with normal controls.

## METHODS

### Subjects

Twenty patients (16 girls and 4 boys) who underwent one-stage surgery with combined open reduction and PPO for unilateral DDH before the age of 4 years between 2006 and 2012 participated in the study at a mean age of 102.5 months (range 73–135 months). All patients were under the care of a single experienced surgeon following the same treatment and rehabilitation programs. The minimum follow-up period was 2 years. The average age at operation was 34.2 months (range 19–47 months) and the mean follow-up time was 68.3 months (range 26–99 months) (Table 1). All the patients were free of pain, joint disorders, and any other muscular or chromosomal disorders. None had undergone any procedure other than the one-stage surgery.

**TABLE 1:**
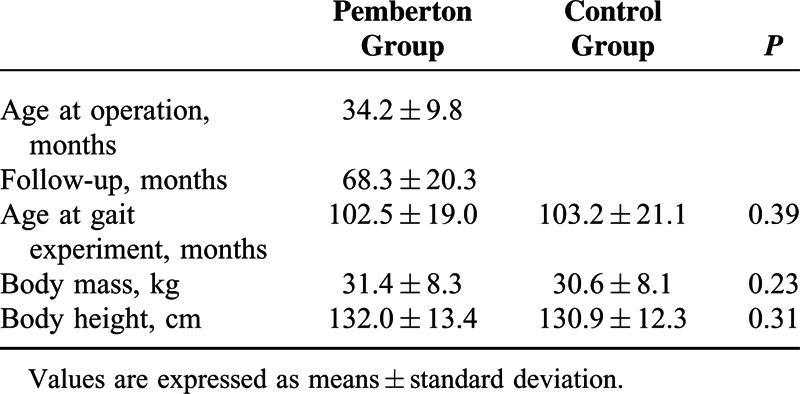
Demographic Characteristic

Radiographic parameters including acetabular index and center-edge angle were measured from a standing anterior–posterior pelvic radiograph preoperatively and at the time of the pedobarographic experiment. The radiographic results were graded using the modified Severin criteria (see Table, Supplemental Content 1, http://links.lww.com/MD/A293, which illustrates the modified Severin criteria for radiographic evaluation).^20^ Clinical data were reviewed retrospectively from the patients’ charts and were evaluated according to the modified McKay criteria (see Table, Supplemental Content 2, http://links.lww.com/MD/A293, which illustrates the modified McKay criteria for clinical evaluation).^21^ Twenty age- and weight-matched healthy subjects were recruited as controls. These subjects had no bone or joint disease, and had no area of pain in the lower limbs that might induce a compensatory abnormal gait pattern. As shown in Table 1, there were no significant differences between the 2 groups in age, height, and body weight.

The study was approved by the Ethical Committee of our university. Parents or guardians signed written informed consent for the patients and controls to participate in the study.

### Instrumentation and Pedobarographic Analysis

**FIGURE 1:**
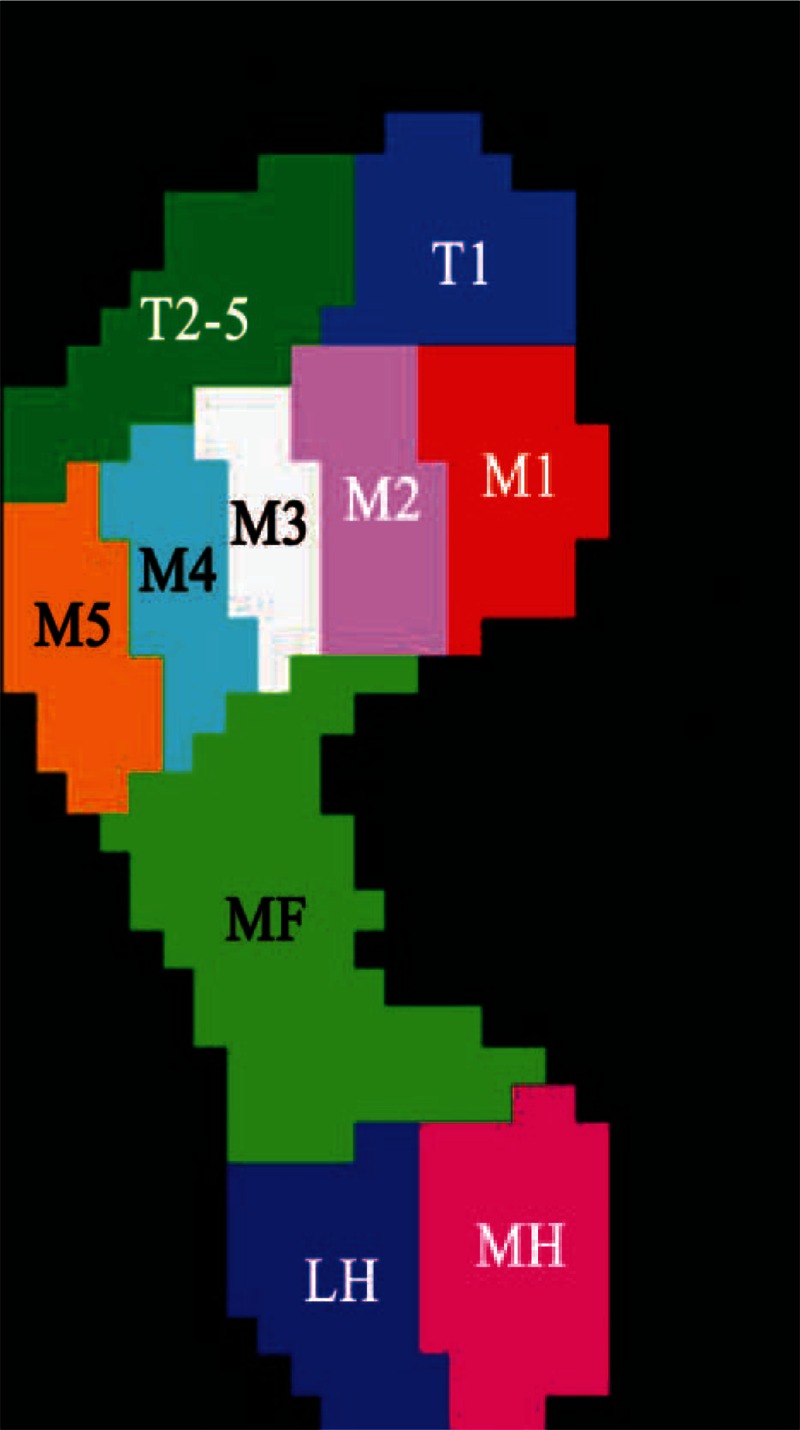
Schematic diagram for the 10 subdivided zones of the foot applied in the current study. The subdivided zones were hallux (T1), toes 2–5 (T2–5), first metatarsal (M1), second metatarsal (M2), third metatarsal (M3), 4th metatarsal (M4), fifth metatarsal (M5), midfoot (MF), medial heel (MH), and lateral heel (LH).

**FIGURE 2:**
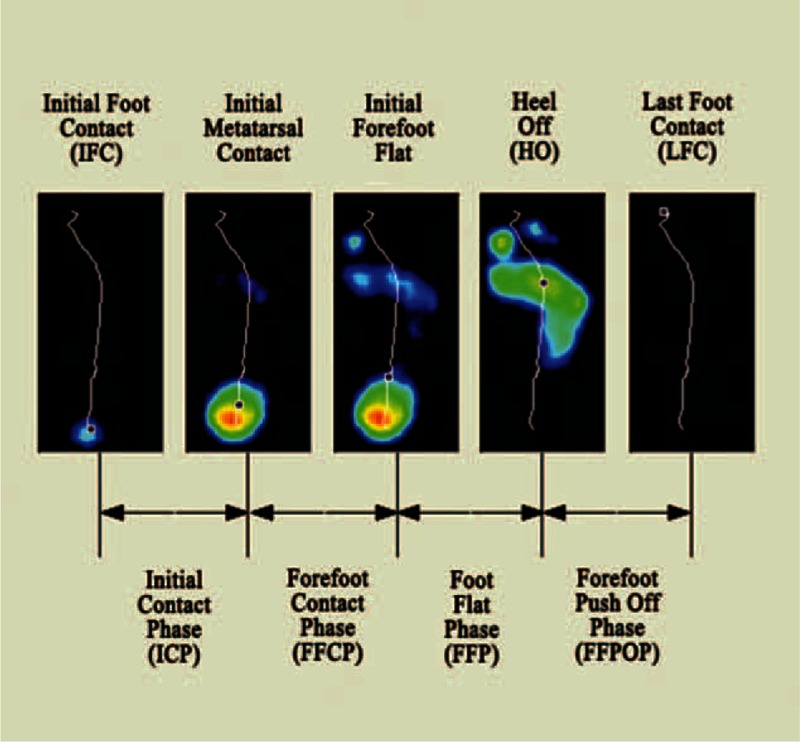
Schematic diagram for the 4 subphases of the stance phase in walking.

Participants were asked to perform pedobarographic tests barefoot at their comfortable walking pace. Plantar pressure was monitored using a 2-m Footscan pressure plate (RSscan International, Olen, Belgium, 2 m × 0.4 m × 0.02 m, 125 Hz and 16,384 sensors). The data were analyzed using Scientific Footscan software (RSscan International). The software automatically divided the foot into 10 masked zones: hallux (T1), toes 2–5 (T2–5), first to fifth metatarsals (M1, M2, M3, M4, and M5), midfoot, medial heel (MH), and lateral heel (LH) (Figure 1). The stance phase was divided into 4 subphases: the initial contact phase (ICP: from the initial foot contact to the initial metatarsal contact with the pressure plate), the forefoot contact phase (FFCP: from the ICP to the point when all the metatarsals were in contact with the pressure plate), the foot flat phase (FFP: from the FFCP until the heel is off the pressure plate), and the forefoot push off phase (FFPOP: from the FFP until the entire foot is off the ground) (Figure 2). The pressure plate was located at the center of a carpet with the same external dimension to provide a “complete platform” 4 m in length to ensure that a minimum of 3 steps were taken before data collection. The platform was disguised with a thin liner of EVA-material to prevent participants adjusting their walking patterns.^22^ Every test comprised a minimum of 5 representative and reliable trials for each foot with intermediate recovery periods of equivalent duration between each trial.

### Data Analysis

Parameters quantifying plantar pressure for each masked zone included peak pressure (PP; in kPa), the force–time integral (FTI; in N s), and contact time (CT) of the 4 subphases in millisecond. FTI was normalized to the total force–time integral of the entire foot (FTI%) and CT was normalized to the stance time of single leg (CT%). In the Pemberton group, the values recorded for each variable were the means of the 5 measurements from the representative and reliable trials. In the control group, each variable was calculated by averaging the data across the limbs.^13^

### Statistical Analysis

Statistical analyses were performed using SPSS software (SPSS 19.0; SPSS Inc, Chicago, IL). The data were normally distributed as tested by the one-sample Kolmogorov–Smirnov test. In the Pemberton group, paired *t* tests were used to detect the differences in PP, FTI%, and CT% between the affected and unaffected limb. Differences between the Pemberton and control groups were analyzed using independent sample *t* tests. One-way analysis of variance with a Dunnett test was performed for multiple comparisons. The significance level was set at 0.05 with a Bonferroni correction for multiple comparisons.

## RESULTS

All hips were concentrically reduced postoperatively. In the affected limb, the acetabular index decreased from a preoperative value of 36.6° ± 8.0° (range 25°–50°) to 16.3° ± 5.8° (range 6°–27°) (*P* < 0.001, paired *t* test) at the time of experiment. The mean final center–edge angle was 37.5° ± 10.1° (range 14°–51°).

Using the modified McKay clinical criteria, 18 hips (90%) were classified as “excellent or good,” 2 hips (10%) were rated as “fair,” and using the Severin radiographic criteria, 14 hips (70%) were classified as “excellent or good,” 6 hips (30%) were rated as ’fair’.

Table 2 shows the comparison of the CT% in the 4 subphases and the total CT in different groups. The mean values of CT% were significantly increased during the FFPOP and significantly decreased during the ICP in the affected limb compared to the control group. In the unaffected limb, values of CT% were lower during the ICP and FFCP and higher during the FFP than in the control group.

**TABLE 2:**
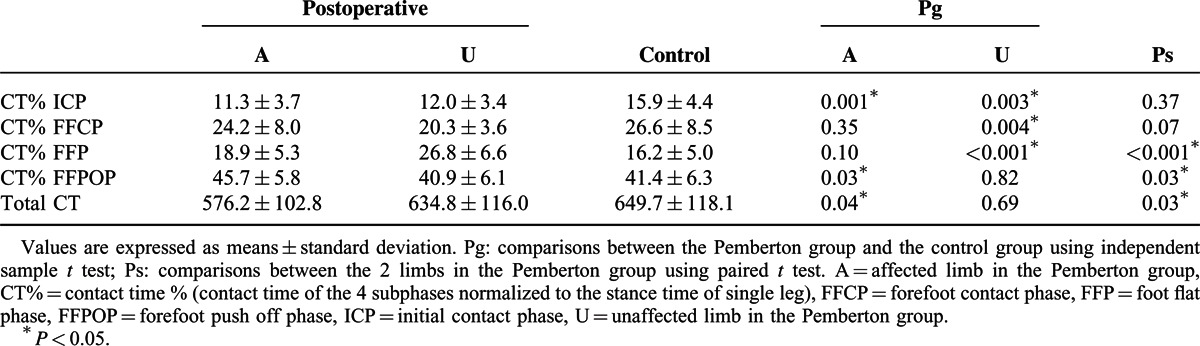
Comparison of Contact Time % in the 4 Subphases and of the Total Contact Time (millisecond) of the Foot

In the Pemberton group, lower values of CT% in the FFP and higher values in the FFPOP were noted in the affected limb than in the unaffected side. In terms of total CT, the mean in the affected limb was significant smaller than in the unaffected side and control group.

The mean values and standard deviations of the PP (kPa) for different zones in the Pemberton and control groups are shown in Table 3. The affected limb showed a higher PP in the M4 and M5 zones than was seen in the control group and the unaffected limb. By contrast, PP in the M1 and M2 zones in the unaffected limb were greater than those in the affected limb. The differences between the unaffected limb and the control group were not statistically significant.

**TABLE 3:**
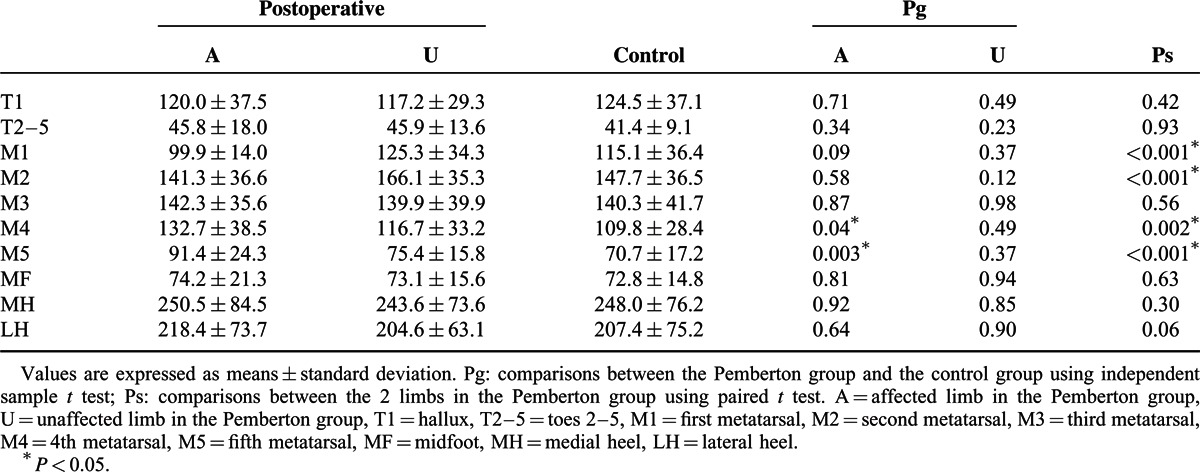
Comparison of the Peak Pressure (kPa) in the 10 Masked Zones

The affected limb showed significantly increased FTI% in the T2–5, M3, M4, and M5 zones, and decreased FTI% in the MH and LH zones compared with values in the control group (Table 4). Significantly lower FTI% in the MH and LH zones and a significantly higher FTI% in the M1 and M2 zones were noted in the unaffected limb compared with corresponding values in the control group.

**TABLE 4:**
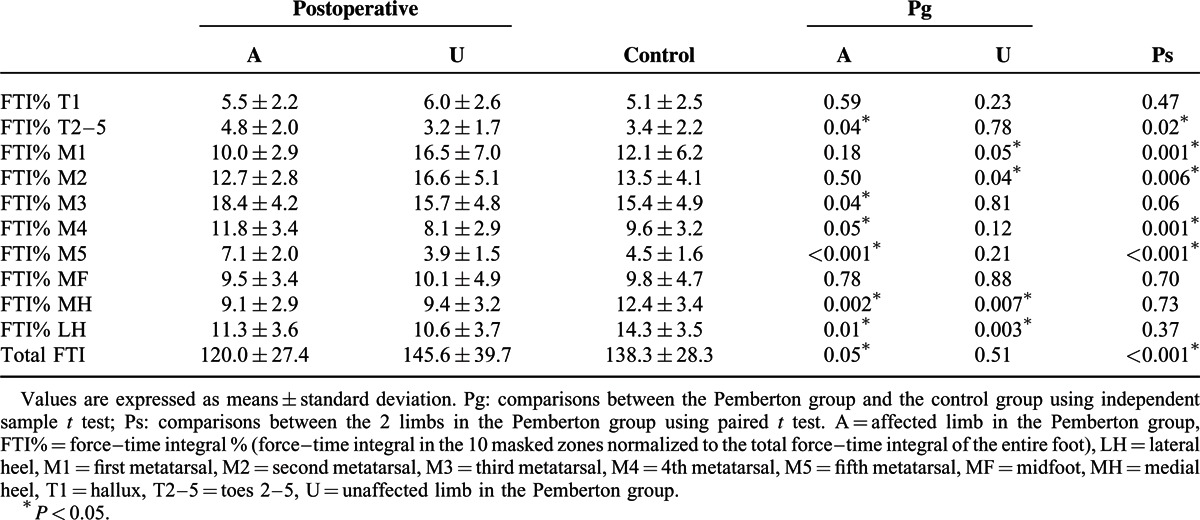
Comparison of Force–Time Integral % in the 10 Masked Zones and of the Total Force–Time Integral (N s) of the Foot

In the Pemberton group, significantly higher values of FTI% in the T2–5, M4, and M5 zones and lower values in the M1 and M2 zones were observed in the affected limb than in the unaffected side. The affected limb also manifested a significantly lower total FTI than values of the unaffected limb and control group (Table 4).

In order to further characterize our population, we stratified 2 groups based on the evaluation results according to the Severin radiographic criteria. The patients who were classified as “excellent or good” (n = 14) were in group 1, those rated as “fair” (n = 6) were in group 2. The 2 groups were compared with controls to assess differences in pedobarographic parameters both in affected limb and unaffected limb. One-way analysis of variance with Dunnett post hoc test was performed for statistical analysis. A Bonferroni adjustment was applied to minimize the chance of false-positive findings (*P* < 0.0125). In group 1, the mean values of CT% were significantly decreased during the ICP in the affected limb compared to the control group. In the unaffected limb of group 1, values of CT% were lower during the ICP and higher during the FFP than in the control group (Table 5). No significant differences in PP were found for either the group 1 or group 2 compared with controls (Table 6). Meanwhile, the affected limb in group 1 showed significantly increased FTI% in the M5 zone, and decreased FTI% in the MH zone compared with values in the control group (Table 7). In group 2, significantly lower FTI% in the MH zone and a significantly higher FTI% in the M1 zones were noted in the unaffected limb compared with corresponding values in the control group (Table 7).

**TABLE 5:**
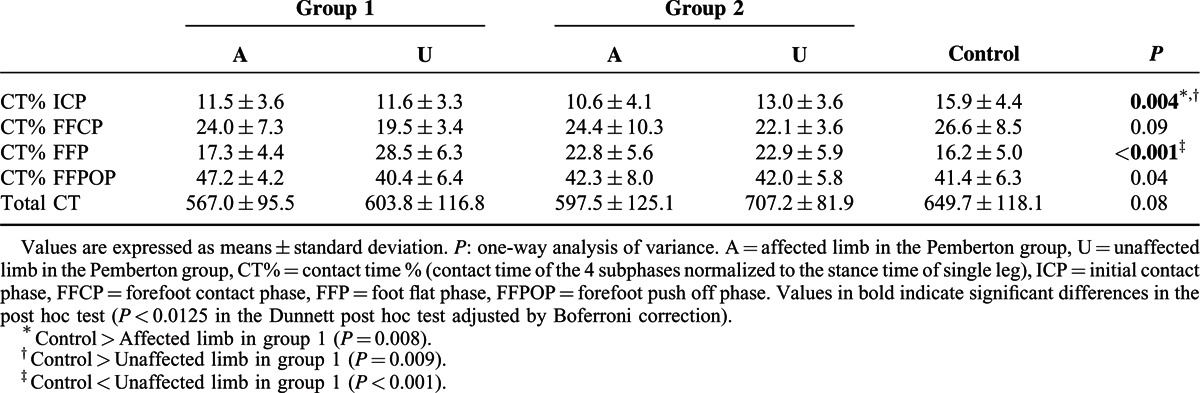
Comparison of Contact Time % in the 4 Subphases and of the Total Contact Time (millisecond) of the Foot: Group 1 (n = 14) and Group 2 (n = 6) Versus Controls (n = 20)

**TABLE 6:**
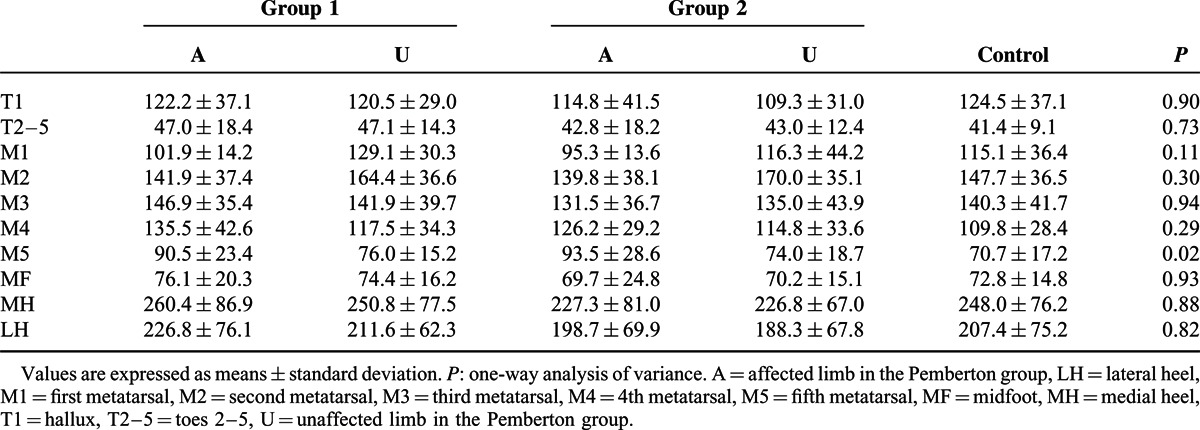
Comparison of the Peak Pressure (kPa) in the 10 Masked Zones: Group 1 (n = 14) and Group 2 (n = 6) Versus Controls (n = 20)

**TABLE 7:**
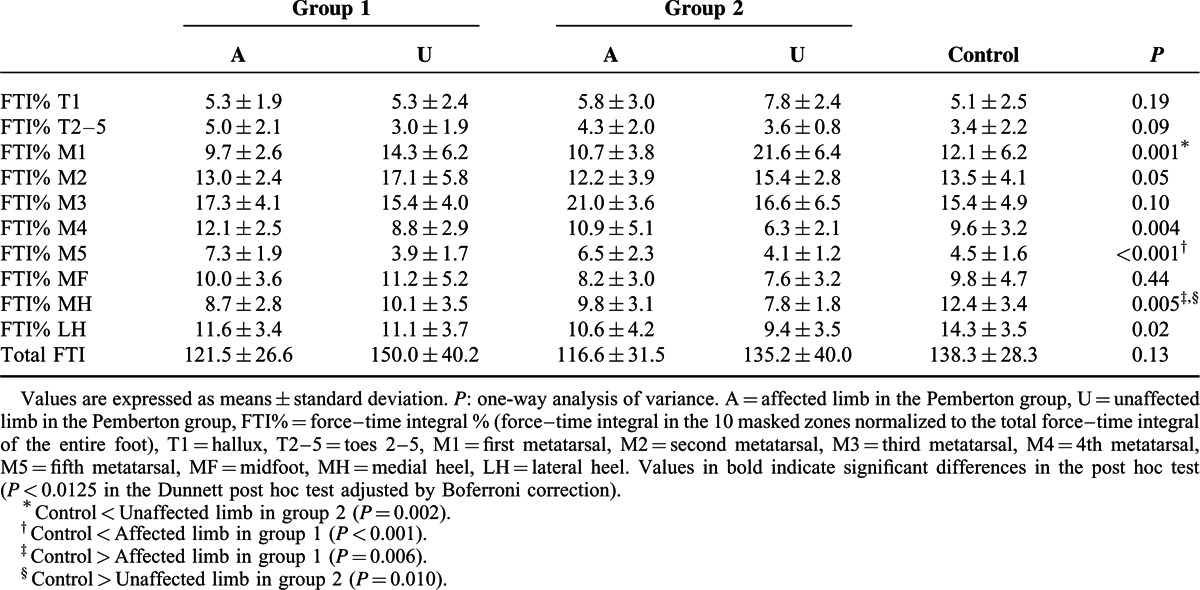
Comparison of Force–time Integral % in the 10 Masked Zones and of the Total Force–time Integral (N·s) of the Foot: Group 1 (n = 14) and Group 2 (n = 6) Versus Controls (n = 20)

## DISCUSSION

Plantar pressure measurements can be used to objectively evaluate postoperative functional behavior of the foot and can help in addressing kinematic and kinetics deviations.^23–26^ These evaluations have been regarded as a useful supplement to clinical and radiologic evaluation.^19^ The present results support the hypothesis that residual deviations in plantar pressures exist in patients who have been treated with PPO for unilateral DDH.

A study in 2011^13^ performed gait analysis on 11 adolescent females after PPO for unilateral DDH. The results indicated lower percentages of stance phase in the gait cycle of the affected limbs. Similarly, in our study, the duration of the stance phase in the affected limb was significantly shorter than in the unaffected limb and control group. We also found that CT% in the FFPOP was greater in the affected limb than those in the unaffected side and control group. These differences may be related to the drawn of the body's center of mass (COM)^12^ and weakness of the hip abductors and flexors^27,28^ of the affected limbs involved in surgery.

When the affected limb enters the stance phase, a decline of the COM in the affected limb may occur because of surgically induced muscular dysfunction combined with compensatory pelvic movement.^12^ When the unaffected side is about to enter the stance phase, the lower COM of the body opposes the movement, causing the affected limb to try to enter the FFPOP more quickly and thereby elevate the COM. Thus, when the affected limb enters the FFPOP, it may make full use of the extension in the knee joint and drive the COM to the level needed for the unaffected limb. Similar postoperative findings have been described by Pedersen et al, ^14^ who concluded subjects walked with a more upright push-off posture with an increased extension in the knee joint.

In the affected side, PP was increased in the M4 and M5 zones compared with normal controls. PP was lower in the affected limb than in the unaffected limb in the M1 and M2 regions but higher than in the unaffected limb in the M4 and M5 zones. These results indicate a load transfer from the medial to the lateral aspect of the forefoot. These changes may be the results of lateral displacement of the action line of the ground reaction force in the affected limb, as previously reported.^12^ They may also be attributed to the increased pelvic hiking and toe-out angle in the affected limb.^12^

Increased PP has been shown to cause foot pain when walking^29,30^ and can also result in lower extremity overuse injuries.^31^ Therefore, monitoring PP may be useful strategy for the early prediction and prevention of the foot pain and injuries in patients who have undergone PPO for DDH.

The FTI of the whole stance phase reflects the integrated effects of force and time, which may indicate the weight-bearing function of the limbs. In the stance phase, the total FTI of the affected limb was significantly lower than values in the unaffected limb and normal controls. This was indicative of a weaker weight-bearing function of the affected limb, probably related to loss of muscle strength (eg, gluteus medius and illopsoas),^27,28^ residual fear resulting from disuse of the affected limb,^32^ altered mechanical properties caused by realignment of the hip,^33^ and intermittent pain. Additionally, the reduced total CT may be responsible for the decreased FTI during the stance phase on the affected side.

In the affected limb, FTI% was reduced in the hindfoot (MH and LH zones) with a compensatory increase in the lateral forefoot (T2–5, M3, M4, and M5 zones) compared with the control group. This was possibly the result of the action line of the ground reaction force shifting forward and laterally.^12^ It may also be related to the shorter ICP and longer FFPOP (Table 2), which would reduce the FTI of the heel and increase that of the forefoot by affecting CT. Thus, postoperative rehabilitation training may be needed for the patients to improve the weight-bearing capacity of the affected side.

In the subgroup analysis, despite our failing to demonstrate same statistical results, we did note a similar trend when group 1 and group 2 were compared with controls. There were no significant differences between patients classified as “excellent or good” and those rated as “fair” according to the modified Severin criteria. However, this result may be biased by the small number of cases and relatively short duration of follow-up, so it was just for reference, further studies with larger sample size and longer follow-up are necessary to clarify this topic.

The patients with unilateral limb disease are known to manifested plantar pressure deviations on the contralateral side.^19,34,35^ In the present study, the pedobarographic parameters for the unaffected limb in the Pemberton group were significantly different to those in the control group. We hypothesized the differences come mainly from an altered gait pattern. The residual gait deviations in the affected side being probably compensated at the knee and ankle joint of the same limb.^12,13^ These changes may induce an asymmetry that is also reflected in the unaffected lower extremity.^19^ Finally, the plantar pressure distribution under the unaffected foot is changed.

Interpretation of our results is potentially limited by lack of the pedobarographic data prior to PPO surgery, which means we were unable to quantify changes in pedobarographic measures from pre- to postoperation. However, the young age of the subjects meant that they were in a rapid growth period of the muscle and skeleton structures, the development of the fat pad and the longitudinal arch would dramatically affect the gait pattern and plantar pressure indicators.^36^ Therefore, the comparison of pedobarographic data between pre- and postoperation would not have provided comprehensive information relevant to evaluating the recovery of the patients.

The young age of subjects meant that it was difficult to get them to repeat the test 5 times without being offered toys or candies to get their cooperation. These factors may have affected the quality of the measurements. However, other pedobarographic studies have been performed using similar experimental methods in even younger children.^23,25,26,36^

Finally, this study is limited by a small number of the participants, which may reduce the generalizability of the research. The adverse effects were minimized by performing large number of measurements to ensure enough qualified data for analyzing.

## CONCLUSIONS

Although the clinical and radiographic outcomes after PPO for unilateral DDH are encouraging, achieving “normalization” of plantar pressure remains challenging. The deviations may be attributed to compensatory efforts of the unaffected limb and other joints of the lower extremity in the affected limb. They may also be attributed to altered muscle, bone, and joint function following PPO, or even psychology factors. Longer follow-up is required to more fully evaluate the effect of these deviations on gait following PPO for DDH.
